# Optimization of the Rheological Properties of Self-Assembled Tripeptide/Alginate/Cellulose Hydrogels for 3D Printing

**DOI:** 10.3390/polym14112229

**Published:** 2022-05-30

**Authors:** Alejandro Hernández-Sosa, Rosa Ana Ramírez-Jiménez, Luis Rojo, Fouzia Boulmedais, María Rosa Aguilar, Miryam Criado-Gonzalez, Rebeca Hernández

**Affiliations:** 1 Instituto de Ciencia y Tecnología de Polímeros (ICTP), CSIC, c/Juan de la Cierva, 3, 28006 Madrid, Spain; alejandrohs@ictp.csic.es (A.H.-S.); raramirez@ictp.csic.es (R.A.R.-J.); mraguilar@ictp.csic.es (M.R.A.); 2 Networking Biomedical Research Centre in Bioengineering, Biomaterials and Nanomedicine, CIBER-BBN, c/Monforte de Lemos 3-5, Pabellón 11, 28029 Madrid, Spain; 3 Institut Charles Sadron (UPR 22), Université de Strasbourg, CNRS, 23 rue du Loess, BP 84047, CEDEX 2, 67034 Strasbourg, France; fouzia.boulmedais@ics-cnrs.unistra.fr; 4 POLYMAT, Department of Polymers and Advanced Materials: Physics, Chemistry and Technology, Faculty of Chemistry, University of the Basque Country UPV/EHU, Paseo Manuel de Lardizabal, 3, 20018 San Sebastian, Spain

**Keywords:** alginate, hydrogels, hybrid polymer inks, peptides, 3D printing

## Abstract

3D printing is an emerging and powerful technique to create shape-defined three-dimensional structures for tissue engineering applications. Herein, different alginate–cellulose formulations were optimized to be used as printable inks. Alginate (Alg) was chosen as the main component of the scaffold due to its tunable mechanical properties, rapid gelation, and non-toxicity, whereas microcrystalline cellulose (MCC) was added to the hydrogel to modulate its mechanical properties for printing. Additionally, Fmoc-FFY (Fmoc: 9-fluorenylmethoxycarbonyl; F: phenylalanine; Y: tyrosine), a self-assembled peptide that promotes cell adhesion was incorporated into the ink without modifying its rheological properties and shear-thinning behavior. Then, 3D-printed scaffolds made of Alg, 40% of MCC inks and Fmoc-FFY peptide were characterized by scanning electron microscopy and infrared spectroscopy, confirming the morphological microstructure of the hydrogel scaffolds with edged particles of MCC homogeneously distributed within the alginate matrix and the self-assembly of the peptide in a β-sheet conformation. Finally, the cytocompatibility of the scaffolds was tested in contact with the MG63 osteosarcoma cells, confirming the absence of cytotoxic components that may compromise their viability. Interestingly, MG63 cell growth was retarded in the scaffolds containing the peptide, but cells were more likely to promote adhesive interactions with the material rather than with the other cells, indicating the benefits of the peptide in promoting biological functionality to alginate-based biomaterials.

## 1. Introduction

Hydrogels are three-dimensional polymer networks with the ability to hold a large quantity of water [1]. They are employed in a great variety of biomedical applications such as 3D scaffolds, mimicking the native extracellular matrix (ECM) for tissue engineering, or as matrices for drug delivery applications because of their swelling and responsive properties, among others [2,3,4]. For such purposes, the scaffolds should be non-cytotoxic, allow for cell adhesion and proliferation, permit nutrient diffusion, and have a degradation period that allows the cells to progressively replace the scaffold with an extracellular matrix [5]. Apart from this, hydrogels are attracting increasing attention in other research fields for the removal of dyes and ions, sensing, or electronic applications [6,7,8]. Since the emergence of 3D printing techniques, leading to shape-defined three-dimensional scaffolds, the development of biocompatible inks constitutes an intensive field of research [9,10,11].

Polymer hydrogels constitute the material of choice for the fabrication of scaffolds through 3D extrusion printing, but there are a limited number of suitable hydrogels that can be formulated as polymer inks. The tuning of their properties remains a challenge [12]. For 3D extrusion printing, polymer inks need to have certain mechanical properties such as a minimum viscosity and a crosslinking capability to retain the structure after printing [13]. The viscosity of the ink, one of the critical parameters studied, increases with the concentration of the polymer as well as with its molecular weight [14,15]. A shear-thinning behavior of the ink is of prime importance for 3D printing [14,15,16]. Indeed, without a shear-thinning behavior, the viscosity of the ink would be too excessively high to be extruded through the nozzle. The yield stress is another critical parameter defined as the critical stress above which the ink will flow and behave as a liquid. Increased yield stress requires higher extrusion pressures, which can negatively affect the cell viability [17]. In addition, it has been found that the ratio between the loss modulus (G”) and the storage modulus (G’) affects the printability [16]. When this ratio (defined as tan δ = G’/G”) is excessively high, the ink presents a fluid-like behavior, and thus the structure collapses after being printed. If it is excessively low, the printed structure would present poor extrudability and a non-uniform filament [16]. Finally, inks must have a fast and almost complete recovery of the solid-like behavior after printing to ensure good shape fidelity [17]. These findings demonstrate the importance of rheology to characterize the viscoelastic properties of the hydrogels in general [3], and in particular, those employed as inks. 

Alginate, a biopolymer extracted from brown algae, is an excellent candidate for 3D extrusion printing due to its tunable mechanical properties, rapid gelation, and low cost [18]. It has been extensively used for bioprinting bone, cartilage, cardiac, and vascular tissues [18,19,20]. However, alginate presents major drawbacks. The first one is its poor mechanical properties, even when cross-linked with divalent ions such as Ca^2+^, Sr^2+^, Zn^2+^, or Fe^2+^ [4,21,22,23]. In this sense, alginate is mixed with other polymers such as gelatin or cellulose to increase the printability and structural integrity of the 3D printed constructs [13,16,24,25]. In particular, cellulose and cellulose derivatives obtained through the chemical modification of cellulose constitute environmentally-friendly materials that have attracted a great deal of attention over the last years due to their characteristics of biocompatibility, biodegradability, and renewability [26,27]. Broadly speaking, cellulose based materials can be classified into two categories depending on the raw source. Bacterial nanocellulose is produced by a special class of acetic acid bacteria as well as by the cell-free enzyme systems and cellulose materials obtained from vegetable biomass that, depending on the method of extraction, can be obtained as cellulose micro- or nanocrystals or cellulose micro- or nanofibers [28]. Cellulose obtained from the vegetable biomass can be extracted from discarded plant products, and thus plays a role in the circular economy [29]. It constitutes an abundant resource with applications such as being a rheology modifier and in 3D bioprinting [30]. A specific form of cellulose is microcrystalline cellulose (MCC), in which cellulose chains arrange into crystals in the micron range. This material has been previously applied to enhance the mechanical and rheological properties of acrylic resins [31], and has other multiple applications in the pharmaceutical, food, or cosmetic industries [32].

The second drawback is that alginate is a bioinert material lacking cell-adhesive moieties [33]. Many efforts have been made to induce an appropriate cellular adhesion in alginate gels. Besides the introduction of gelatin or collagen in the ink [16], one strategy is to graft different cell adhesive peptides to alginate [34,35]. Recently, Criado-Gonzalez and coworkers [36] showed that the incorporation of a self-assembled phosphorylated tri-peptide, Fmoc-FFY (Fmoc: 9-fluorenylmethoxycarbonyl; F: phenylalanine; Y: tyrosine), within the polyethylene glycol [37] or hyaluronic acid hydrogels allowed for the promotion of fibroblast cell adhesion [36,38]. Moreover, this peptide exhibited antibacterial and anti-inflammatory properties [39,40].

The main purpose of our study was to determine the printability of Fmoc-FFY tripeptide/alginate/cellulose hydrogel inks and its potential to obtain non-cytotoxic cell scaffolds that were able to promote cell–material interactions in vitro. First, shear-thinning alginate hydrogels were obtained by pre-crosslinking with CaCl_2_. Then, the incorporation of microcrystalline cellulose (MCC) allowed for an increase in the viscosity of the ink and to retain its shape after printing. For such purposes, a deep rheological study was carried out as well as the filament and layer stacking tests. To form the self-assembled Fmoc-FFY peptide network within the alginate ink, Fmoc-FFpY, a precursor peptide, was incorporated with alkaline phosphatase (AP), an enzyme that is able to cleave the phosphate group of Fmoc-FFpY. A lab-made extrusion printer was employed to print scaffolds that were further characterized by scanning electron microscopy (SEM) and infrared spectroscopy. Finally, non-adherent MG63 osteosarcoma cells were employed for the in vitro tests due to their ability to grow forming clusters into hydrogels driven by cell-to-cell interactions or to growth, individually establishing focal adhesive filopodia when cultured on adhesive-promoting biomaterials.

## 2. Materials and Methods

### 2.1. Materials

Alginic acid sodium salt (Alg) of low viscosity (Brookfield Viscosity 4–12 cps at a concentration of 1% in H_2_O at 25 °C, according to the manufacturer) was extracted from brown algae and microcrystalline cellulose (MCC) extracted from cotton linters (particle size in the range of 15 to 70 microns, Figure S1 in the Supplementary Materials) and were purchased from Sigma-Aldrich (Saint Louis, MO, USA) and used as received. Fmoc-FFpY (86% purity) was purchased from PepMic. Phosphate-buffered saline (PBS), calcium chloride, and the enzyme alkaline phosphatase (AP) were purchased from Sigma-Aldrich and used as received. 

### 2.2. Ink Preparation

#### 2.2.1. Preparation of the Pre-Crosslinked Alginate Inks with CaCl_2_

Alginic acid sodium salt was dissolved in PBS (pH 7.4) at a concentration of 14% *w*/*v*. Then, the 14% *w*/*v* alginate solution was mixed with an aqueous ionic pre-crosslinker solution of CaCl_2_ (60 mM) at different volumetric ratios (alginate:CaCl_2_) of 2.5:1, 2:1, and 1.5:1 using the dual syringe approach. Later, PBS was added to the mixture until a final alginate concentration of 8% *w*/*v* was reached. Subsequently, the inks were homogenized by passing them through a 25G (0.25 mm diameter) nozzle three times and refrigerated at 4 °C for 48 h prior to testing.

#### 2.2.2. Modulation of the Pre-Crosslinked Inks Viscosity by Incorporation of Microcrystalline Cellulose and Printability Tests

MCC was incorporated in the powder at different concentrations of 1%, 10%, 20%, and 40% (*w*/*v*) in the 8% *w*/*v* alginate inks pre-crosslinked with 60 mM CaCl_2_ to increase their viscosity and improve the printability properties.

Then, the resulting inks were tested for printability by the filament test and the layer stacking test [41]. These tests were performed with a bioprinter using smooth flow tapered tips with a 0.25 mm diameter.

#### 2.2.3. Incorporation of the Fmoc-FFpY Peptide into the Ink Formulation

The Fmoc-FFpY peptide, at a concentration of 2 mg/mL, and alkaline phosphatase (AP), at a concentration of 2 mg/mL, were incorporated into the Alg_MCC_40_ ink formulations, giving rise to Alg_MCC_40__FFY ink.

**Scheme 1:**
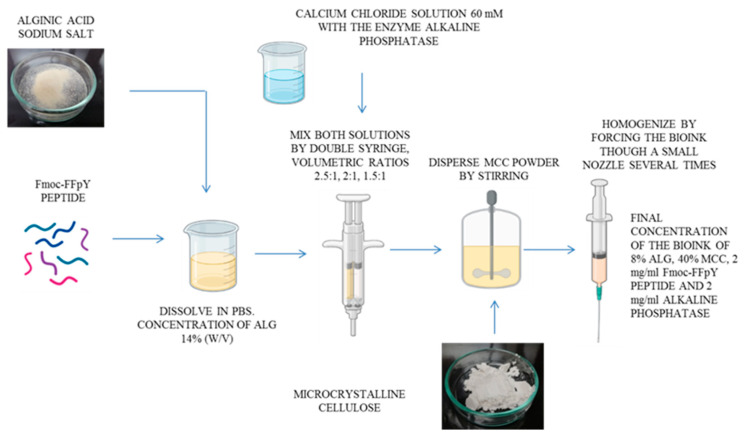
The procedure employed for the preparation of alginate-based ink formulations reported herein.

A schematic representation of the whole procedure employed for the preparation of the inks is provided as Scheme 1. 

### 2.3. Rheological Characterization of the Inks

The rheological properties of the alginate–cellulose inks were measured with an AR-G2 rheometer (TA instruments^®^, New Castle, DE, USA) at 37 °C. Viscosity tests were performed by varying the shear rate from 0.001 s^−1^ to 100 s^−1^ using a cone-plate geometry of 40 mm and a gap of 58 µm. A mathematical model, known as the Power Law equation, was fitted to the viscosity curves (Equation (1)) [42]:

(1) $$\eta =K\;{\dot{\gamma }}^{n-1}$$

where η is the viscosity; $\dot{\gamma }$ is the shear rate; and *K* and *n* are the fitting parameters. The parameter *K* represents the viscosity of the ink at a shear rate of 1 Pa/s. The parameter *n* is inversely proportional to the shear-thinning behavior of the material, and low values of *n* (*n* < 1) indicate a shear thinning behavior.

The linear viscoelastic region and yield strain were determined by oscillatory strain sweeps ranging from 0.01% to 1000% strain at a constant frequency of 1 Hz. A storage modulus at 1 Hz was obtained from oscillatory frequency sweeps ranging from 0.01 Hz to 100 Hz at 0.1% strain. Finally, the storage modulus recovery was determined by dynamic oscillatory strain cycles at 1 Hz. Each cycle consisted of a 0.1% strain deformation (in the linear viscoelastic region) for 200 s followed by a 100% strain deformation (above the ink yield stress to resemble extrusion in the 3D bioprinter) for 200 s. This cycle was repeated three times. All oscillatory tests were carried out using a parallel cross-hatched plate geometry with a 20 mm diameter and a gap of 1500 µm. Measurements were carried out in triplicate.

### 2.4. 3D Printing of Alginate Inks

#### 2.4.1. Transformation of a Filament Extrusion 3D Printer into a Bioprinter

A Creality Ender 3 Pro 3D printer was transformed into a 3D bioprinter. First, all of the pieces needed for the printer modification were designed using the SolidWorks Premium 2019 SP3.0 software (Dassault Systemes^®^, Velizy-Villacoublay, France) and printed with a Creality Ender 3 Pro 3D printer using polylactic acid (PLA). Then, the printed pieces were mounted on the printer by additionally employing a threaded rod with a 8 mm diameter, two 8 mm diameter smooth rods, standard screws, and two linear bearings (SCS8UU). The step motor that extrudes the syringe is the extruder motor that came with the original 3D printer. The transformed 3D bioprinter can print with syringes of 1, 3, and 5 mL and extrude up to 3 mL of ink per minute at printing speeds between 0.1 and 180 mm/s. 

#### 2.4.2. Optimization of Printing Parameters

A rectangular prism with the dimensions of 40 × 25 × 1 mm was designed with the Creality Slicer software v4.2 (Creality^®^, Shenzen, China), giving rise to a zigzag pattern that was used to assess the printing fidelity. The parameters applied during the slicing process were a 3% zigzag infill density and a layer height of 1 mm. All inks were printed with the modified 3D printer using smooth-flow tapered tips of 25G (0.25 mm diameter, Nordson^®^, Westlake, OH, USA). Different flow rates (0.015, 0.03, and 0.045 mL/min) and printing speeds (1, 2.5, and 5 mm/s) were tested. The optimal printing parameters were selected according to the spreading ratio (SR), a representative measurement of the printing fidelity [43]. The spreading ratio is the quotient between the printed filament diameter and the nozzle diameter (Equation (2)):

(2) $$\mathrm{SR}=\frac{\mathrm{Printed}\;\mathrm{filament}\;\mathrm{diameter}}{\mathrm{Nozzle}\;\mathrm{diameter}}$$

Once the extrusion flow rate and printing speed parameters were adjusted for each ink, cylinders with a 10 mm diameter and 5 mm total height were printed with different layer heights in order to optimize this parameter. Cylinders were sliced using a 25% linear infill density, a wall thickness of 0.2 mm, and layer heights of 0.5, 0.75, 1.0, and 1.25 mm. 

#### 2.4.3. 3D Printing of Alginate Scaffolds

Cylinders with a 12 mm diameter and 2 mm height were printed at the optimal printing conditions determined previously. Cylinders were sliced using a 25% linear infill density and a wall thickness of 0.2 mm. After printing, the samples were immersed in 100 mM CaCl_2_ for crosslinking overnight.

### 2.5. Characterization of the Printed Scaffolds

#### 2.5.1. Morphological Characterization by Scanning Electron Microscopy

Scanning electron microscopy (SEM) observations were performed on a Phillips XL30 with a tungsten filament at a voltage of 25 kV. Previously, the samples were coated with Au in an SC7640 High-Resolution Sputter Coater (Quorum Technologies). 

#### 2.5.2. Water Content

The content of water inside the scaffolds was determined by measuring the mass changes of the scaffolds in the wet state (*m_wet_*) after printing, and in the dried state (*m_dry_*) by freeze-drying the scaffolds and using the following equation:

(3) $$Water\left(\%\right)=\frac{m_{wet}-m_{dry}}{m_{wet}}\times 100$$

The results are given as the mean ± sd (*n* = 5).

#### 2.5.3. Fourier Transform Infrared Spectroscopy (FTIR)

The ATR-FTIR spectra were acquired from dried inks in a Perkin Elmer Spectrum One spectrometer. For the visualization of peptide self-assembly by itself, Fmoc-FFpY and AP were mixed in PBS and an aliquot of the solution was subsequently dried. The spectra were recorded in the range of 4000–400 cm^−1^ with a resolution of 4 cm^−1^ and 20 scans per sample.

### 2.6. In Vitro Cytotoxicity Tests

#### 2.6.1. Cell Culture

The MG-63 (ECACC 86051601, passage 13) cells were cultured in basal medium consisting of Dulbecco’s modified Eagle’s medium (DMEM)—high glucose enriched with 110 mg/L of sodium bicarbonate and supplemented with 10% *v*/*v* of fetal bovine serum (FBS, Gibco), 1% MEM Non-Essential Amino Acids (MEM NEEAA-100X), 200 mM L-glutamine, 100 units/mL penicillin, and 100 mg/mL streptomycin. Cells were maintained under a humidified 5% CO_2_ atmosphere at 37 °C in a Thermo-Fisher incubator model 3541.

#### 2.6.2. Cytotoxicity Assay

Scaffolds for the cytotoxicity assays (*n* = 3) were printed and immersed in 70% ethanol for sterilization for 90 min. Afterward, the ethanol was removed, and the samples were rinsed and washed with sterile PBS for 15 min under gentle shaking. Then, the washing procedure was repeated, and the samples were immersed in 4 mL PBS (containing 100 units/mL penicillin and 100 mg/mL streptomycin) at 37 °C under gentle shaking in sterile conditions. After 24 h, extracts of 3 mL were taken and replaced with fresh PBS for another 24 h. The MG63 cells were seeded in 96-well plates (Fisher Scientific, Waltham, MA, USA) at a density of 9 × 10^4^ cells/mL in 100 µL of basal medium and incubated for 24 h. Afterward, the media were removed and replaced by 100 µL of 50:50 *v*/*v* mixtures of the basal medium and PBS extracts (*n* = 8). After 24 h of incubation, the media was removed and 100 µL of the Alamar Blue (AB, BIO-RAD BUF012B) solution prepared in warm DMEM–low glucose phenol red-free (10% *v*/*v*) (Gibco) was added to each well and the plates were incubated at 37 °C for 3 h. Then, the fluorescence was measured at 590 nm after excitation at 560 nm using a microplate reader (Synergy HT). Relative cellular viability was calculated after subtracting the fluorescence of the blank (AB solution in empty wells) from the fluorescence of the samples and normalizing to the data of the untreated cultures, which were given an arbitrary value of 100.

#### 2.6.3. Cell Adhesion and Proliferation

Sterile-gel scaffolds were placed in 48-well plates and washed once with 1 mL of PBS for 5 min and twice with 1 mL of DMEM. Subsequently, 50 µL of MG-63 cells (4 × 10^4^ cells) were seeded on top of the gels and subsequently incubated at 37 °C and 5% CO_2_ for 40 min. Afterward, 500 µL of culture medium was added and cultured for 24 h for cell adhesion. The adhesion and metabolic activity of the cells in the gel scaffolds were determined by direct assay with the Alamar Blue^®^ reagent. After 1, 3, and 7 days of culture, the scaffolds were transferred to a new 48-well plate and 500 μL of 10% (*v:v*) Alamar Blue^®^ solution (InvitroGen^®^, Waltham, MA, USA) in the medium without phenol red was added to each well. After 3 h of incubation, 100 μL of Alamar Blue^®^ solution was transferred to a 96-well cell culture plate (Greiner Bio-one, Frickenhausen, Germany) in triplicate. The fluorescence was measured at an excitation wavelength of 530/25 nm and at an emission wavelength of 590/35 nm using Biotek Synergy HT plates (Biotek Instruments, Winooski, VT, USA). The diagrams include the mean ± SD (*n* = 5). The ANOVA of the results was performed by comparing the time points with each other at significance levels of * *p* < 0.05.

The morphology of the cells directly in contact with the hydrogels was visualized by epifluorescence microscopy and SEM. The gels were washed with 1 mL of PBS and the cells were fixed/permeabilized with 3.7% *w*/*v* paraformaldehyde (PFA) in PBS for 1 h. The PFA was removed, and the samples were washed twice with 1 mL of PBS. After removing the PBS, 200 μL of Triton 0.05% *w*/*v* in PBS was added and kept in contact for 20 min at room temperature. Later, the samples were washed twice with 1 mL of PBS and brought into contact with 100 μL of Hoechst (dilution 1:1000 from 10 mg·mL^−1^ in PBS) plus 100 μL of phalloidin–rhodamine (dilution 1:100 in PBS from stock) and incubated for 40 min in the dark. Subsequently, the samples were washed once with 400 μL of PBS and then with 1 mL of Milli-Q water. After that, 200 μL of Tween 20 (0.02% *v*/*v* in PBS) was added and incubated for 5 min in the dark. Finally, the samples were washed with 1 mL of Milli-Q water, sliced into 1-mm thick discs and observed by fluorescence microscopy using a Nikon Eclipse TE2000-S epifluorescence microscope with a 20× objective. Afterward, the same samples were dried at room temperature and coated for SEM observation as described above.

## 3. Results and Discussion

### 3.1. Rheological Characterization of Alginate Inks Pre-Crosslinked with CaCl_2_

**Figure 1:**
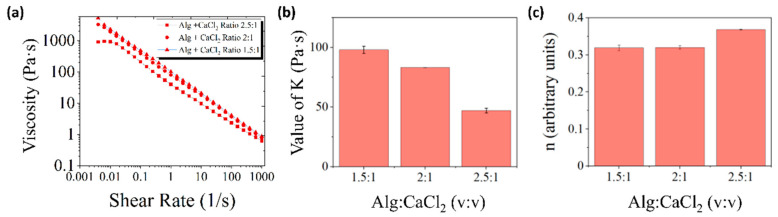
(**a**) The flow sweep curves of the inks with different alginate:pre-crosslinker ratios. Values of (**b**) *K* and (**c**) *n* obtained from the fitting of the viscosity curves to the Power Law equation.

Viscosity is one of the critical factors for 3D printing. A high viscosity value is favorable for 3D printing since it avoids droplet formation and prevents the collapse of the final structure [44]. Nonetheless, if the ink is too viscous, it results in a clog of the printing head and therefore failure of the printing process. A shear thinning behavior, which allows the ink extrusion through the nozzle, is also required for 3D printing. The viscosity of the pre-crosslinked alginate inks were evaluated by rheology at different alginate: CaCl_2_ ratios, with 60 mM CaCl_2_. All ink formulations presented a shear thinning behavior as the viscosity decreased with the shear rate (Figure 1a).

Viscosity curves were fitted to the Power Law model (Equation (1)) and the parameters *K* and *n* were calculated. The parameter *K* is simply the viscosity at a shear rate of 1 s^−1^ and the parameter *n* is related to the slope of the viscosity curve. Inks with a higher amount of pre-crosslinker solution have higher viscosities, as proven by a greater value of *K* (Figure 1b), while presenting an enhanced shear thinning behavior with a lower value of *n* (Figure 1c). Therefore, the selected ratio of alginate:CaCl_2_ was 1.5:1 for the 3D printing tests.

### 3.2. Modulation of the Viscosity by Incorporation of Microcrystalline Cellulose within the Pre-Crosslinked Alginate Ink

**Figure 2:**
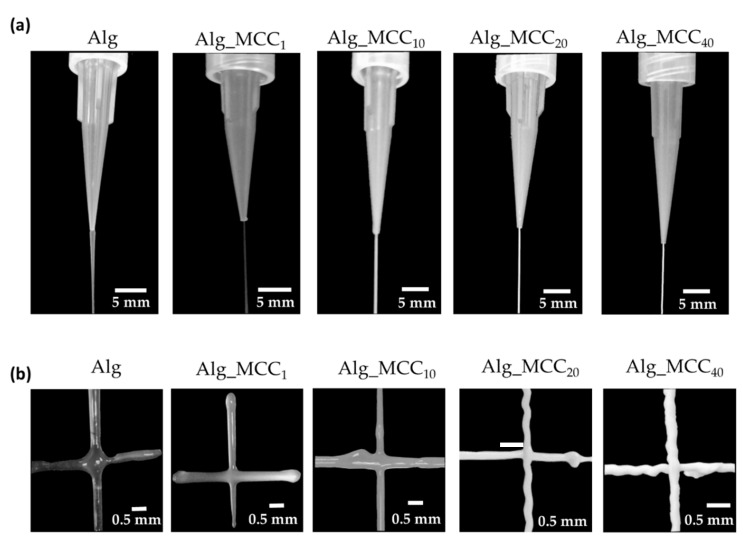
(**a**) The filament test and (**b**) layer stacking test of the different alginate–cellulose ink formulations.

Extrusion 3D printing is a versatile tool that allows for the creation of three-dimensional structures with a wide range of materials. Two requirements must be fulfilled by the ink to be used in 3D printing: (i) the formation of a filament when extruded from a nozzle, is the most important ones, and (ii) the buildup of stable 3D structures by stacking multiple printed layers without fusing [41]. In this regard, the pre-crosslinked alginate inks were not able to fulfill this second requirement. Thus, microcrystalline cellulose (MCC) was added to the formulation to increase the viscosity and mechanical properties of the ink. Figure 2a shows the results of the filament test for several ink formulations containing different MCC concentrations. At the lowest MCC concentration (1% *w*/*v*), the Alg_MCC_1_ ink was too liquid and unable to form a stable filament when extruded, whereas a continuous filament was obtained for the MCC concentrations higher than 10% *w*/*v*. In the case of the layer stacking test, which consists of printing one filament on top of the other, forming a cross without fusing, only the Alg_MCC_20_ and Alg_MCC_40_ inks could stack multiple layers without fusing (Figure 2b). The Alg_MCC_40_ ink was selected for further 3D printing experiments because of its enhanced ability to retain its shape over time after printing.

### 3.3. Rheological Characterization of the Optimized Alginate-Cellulose Ink

**Figure 3:**
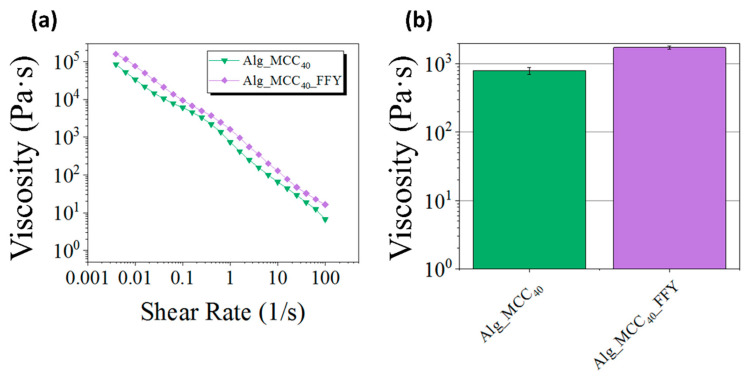
(**a**) The flow sweeps and (**b**) viscosity at a shear rate of 1 s^−1^ of the different ink formulations containing MCC and with/without the Fmoc-FFY peptide network.

After optimization of the alginate–cellulose ink using the filament test, the viscosity of the alginate–cellulose ink was measured by a flow sweep. Moreover, the Fmoc-FFY network was self-assembled within the Alg_MCC_40_ ink by adding 2 mg/mL Fmoc-FFpY and 2 mg/mL AP to obtain the Alg_MCC_40__FFY ink. A shear thinning behavior of the Alg_MCC_40_ and Alg_MCC_40__FFY inks was observed with a decrease in the viscosity as the shear rate increased (Figure 3a). Shear thinning behavior is necessary for extrusion-based printing applications in order to be able to extrude the hydrogel without clogging and achieve structural recovery and integrity post-printing for the printed scaffolds [45]. In comparison to the alginate ink, the viscosity at a shear rate of 1 s^−1^ (*K*) was increased by almost 1-fold with the incorporation of MCC (Figure 1b and Figure 3b), whereas the incorporation of the Fmoc-FFY peptide network did not produce any significant change in the viscosity obtained. The viscosity values obtained were in agreement to the values reported in the literature for alginate–cellulose inks with values of viscosity at a shear rate of 1 s^−1^ in the range of 1000–1500 Pa·s. It is important to note that these values were obtained on formulations containing nanofibrillated cellulose or nanocrystalline cellulose and not with MCC, as reported herein [13,46].

**Figure 4:**
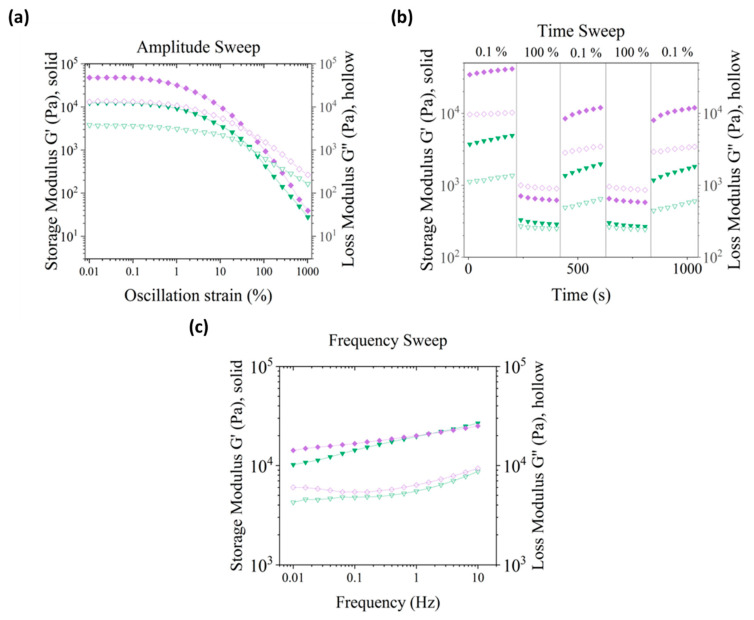
(**a**) The strain sweeps, (**b**) dynamic strain tests alternating 0.1% and 100% strain at short times, and (**c**) frequency sweeps for the inks: Alg_MCC_40_ (▼) and Alg_MCC_40_ _FFY (♦).

Next, the viscoelastic properties of the inks were measured by dynamic oscillatory rheology at 1 Hz. The linear viscoelastic regime was determined by the amplitude sweeps (Figure 4a), allowing us to obtain the yield strain, defined as the minimum strain needed to apply for the ink to start flowing (G” > G’). This value is related to the pressure needed for pneumatic-based 3D printing, and, in our case, to the force needed by the step motor to push the piston of the syringe in piston-based printing. The yield strain was found to be within the experimental error for the Alg_MCC_40_ ink and the Alg_MCC_40__FFY ink being ~45% for both. The self-healing properties after printing were studied by dynamic step strain amplitude tests (Figure 4b) by varying the strain between 0.1% and 100% at short times (200 s). At low strains (0.1%), the inks showed the characteristic rheological behavior of a gel (G’ > G”). After applying a high strain (100%), G’ drastically decreased and a liquid-like state was reached (G” > G’). Then, a gel-like behavior was recovered once the samples were subjected to low strain (0.1%). Such behavior was observed even after two high–low strain repeated cycles and was associated with a self-healing behavior and thee injectability properties of the inks. Next, frequency sweeps were performed in the linear viscoelastic region at 0.1% strain (Figure 4c). The storage modulus (G’) was higher than the loss modulus (G’) for both samples, which means that they showed the rheological behavior characteristic of a gel. From these frequency sweeps, the values of the storage modulus and Tan δ (Tan δ = G”/G’) at 1 Hz were calculated. The presence of the peptide within the alginate ink does not influence the obtained storage modulus of G’ = 22 ± 3 kPa for the Alg_MCC_40_ ink and G’= 24 ± 3 kPa for the Alg_MCC_40__FFY ink. These moduli are in the range of those reported in the literature for alginate–cellulose inks that report values from the order of hundreds of Pa [24] to the order of several kilopascals [13,46]. Likewise, the value of the Tan δ = 0.3 was found to be within the experimental error for both inks, with this value of Tan δ in the printable range as defined by other studies (0.25–0.45) [16].

### 3.4. Optimization of Printing Parameters

Once the Alg_MCC_40_ ink had been formulated to fulfill the filament and layer stacking tests, the operational printing parameters (i.e., flow rate, printing speed, and the height of the layers) were optimized to obtain 3D scaffolds with a well-defined structure using the lab-made 3D printer.

**Figure 5:**
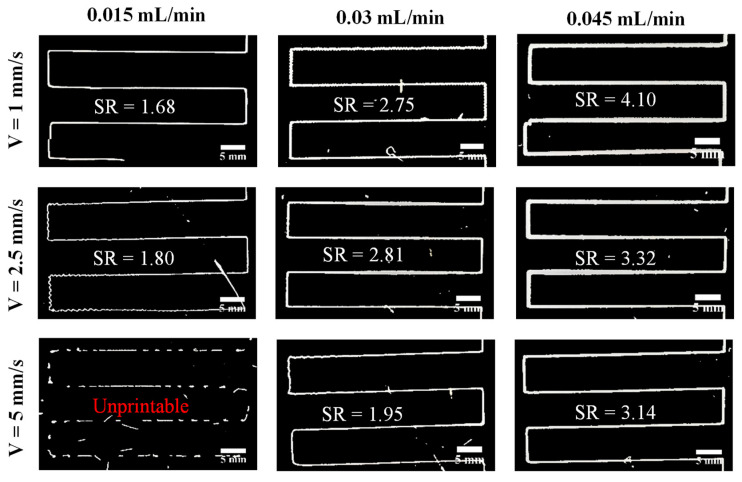
The optimization of the printing speed and flow rate for the Alg_MCC_40_ ink. The spreading ratio of the printed shape was calculated using the ImageJ 1.52a software (Wayne Rasband, National Institutes of Health, Bethesda, MD, USA).

First, the flow rate and printing speed were optimized by printing a zigzag pattern by varying the parameters above-mentioned (Figure 5). For an optimal printing process, the printing speed and flow rate should minimize the spreading ratio (SR) (Equation (2)); at the same time, the printed pattern should be continuous and uniform. Increasing the flow rate resulted in a thicker filament (higher SR). In contrast, an increase in the printing speed gave rise to a thinner filament (lower SR). From the results in Figure 5, a print speed of 2.5 mm/s and a flow rate of 0.015 mL/min were selected for the ink to minimize the spreading ratio and produce a continuous shape. Apart from that, the height of the layers needs to be optimized. For this purpose, cylinders with a 10 mm diameter and a final height of 5 mm were printed by varying the height of each printed layer from 0.5 mm to 1.25 mm. For the Alg_MCC_40_ ink, the best quality results were obtained with a layer height of 0.5 mm.

### 3.5. Morphological Characterization of 3D Printed Scaffolds

**Figure 6:**
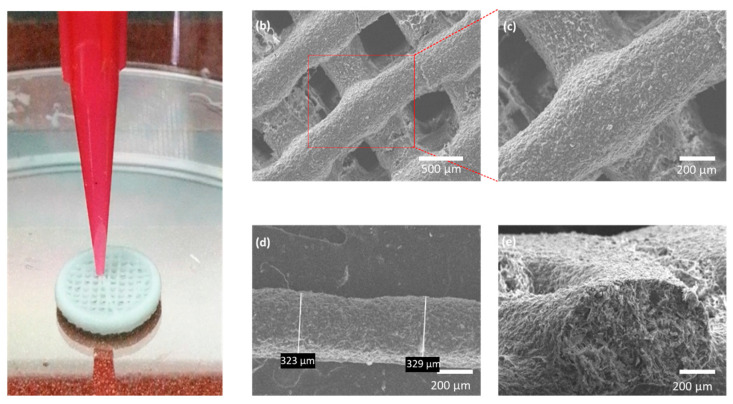
A picture of a representative 3D printed scaffold with the Alg_MCC_40_ ink (**a**) and SEM images for this scaffold: (**b**) Top-view, (**c**) zoom-in of the area marked with the red rectangle, (**d**) single printed filament with measurements, (**e**) cross-section of printed mat filament.

The morphology of the printed scaffolds using the Alg_MCC_40_ ink and a 25% infill percentage of the solid material were observed at a microscopic scale by SEM (Figure 6). The printed scaffolds showed a high shape fidelity, which was successfully retained after printing (Figure 6b). The zoom-in of the area marked by the red rectangle (Figure 6c) allowed for better observation of the section where two filaments from different layers were crossed without fusing, as desired for 3D printing. The printed filaments showed an approximate diameter of 330 μm. The average pore size of the scaffold was 0.47 ± 0.05 mm (Figure S2). It has been found in other studies that the pore size plays a role in the extracellular matrix formation and tissue regeneration (i.e., sizes larger than 100 μm are favorable for mineral formation in bone regeneration) [47]. Additionally, we investigated the internal structure of the freeze–dried scaffold by visualizing the cross-section (Figure 6d). It showed a homogeneous fibrillar morphology, similar to that observed for the freeze–dried 3D printed alginate/chitosan scaffolds [48]. It is important to note that the height of the scaffolds was different depending on the layer height. The best quality results were obtained with a layer height of 0.4 mm. A higher layer height prevented the proper adhesion of the filament to the deposition plate (Figure S3). The total height of the scaffold should match that of the 3D model, which in this case was 5 mm. The outer wall of the cylinder consisted of a single filament. A picture of the 3D model and the final scaffold result is included to compare the shape fidelity. In red is the outer wall and in orange is the infill. After crosslinking with CaCl_2_, no significant contraction or expansion of the scaffold was observed (Figure S4). The percentage of water inside the crosslinked scaffolds printed with the Alg_MCC_40_ ink and with the Alg_MCC_40__FFY ink was found to be ~71% for both cases. To summarize, the printing and rheological properties including the filament thickness and printing fidelity of the alginate-based inks reported herein are similar to those reported in the literature for similar alginate-based inks including alginate/chitosan hydrogels [49] and hybrid hydrogels that constituted of a mixture of alginate, methyl cellulose, trimethyl chitosan, and silicate glasses [47].

**Figure 7:**
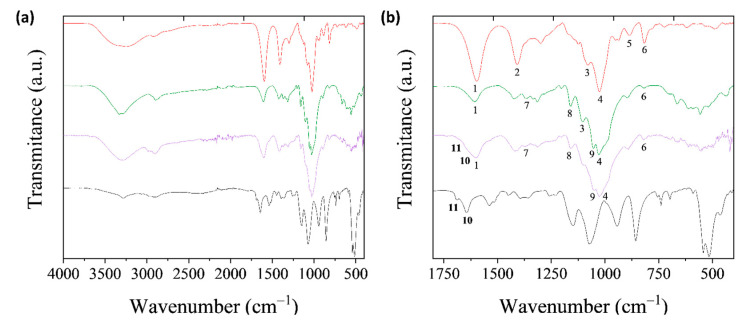
(**a**) The ATR-FTIR spectra of the inks: Alg (red curve), Alg_MCC_40_ (green curve), and Alg_MCC_40__FFY (purple curve), Fmoc-FFY (black curve) and (**b**) zoom-in of the area of interest. Note that the numbers indicate the relevant peaks.

ATR-FTIR spectroscopy was employed to study the chemical structure of the inks (Figure 7a). In the region of 4000–2000 cm^−1^, two bands common to all polysaccharides appeared, a broad band at 3260 cm^−1^ due to the O–H stretching vibrations, and a peak at 2926 cm^−1^ was attributed to the C–H stretching vibrations [50]. To obtain a better insight into the specific signals of every component of the ink, the region oof 1760–400 cm^−1^ was analyzed in detail (Figure 7b).

Six characteristic peaks (numbered from 1–6) were found in the alginate spectrum (red curve) in this region [51]. Peak 1 at 1600 cm^−1^ corresponded to the carboxylate group (COO^−^) and peak 2 at 1407 cm^−1^ to the C–OH deformation vibration. Peaks 3 (at 1083 cm^−1^) and 4 (at 1024 cm^−1^) may be assigned to the C–O and C–C stretching vibrations, respectively [51]. Finally, bands at 878 cm^−1^ and 817 cm^−1^ (peaks 5 and 6, respectively) were characteristic of β-mannuronic acid residues of sodium alginate. When MCC was added to the ink formulation (green curve), new peaks (numbered from 7 to 9), which were characteristic of MCC, appeared. The small peak at 1372 cm^−1^ (peak 7) was due to the CH deformation and the band at 1160 cm^−1^ corresponded to the C–O–C asymmetric stretch vibrations (peak 8) [52,53]. A last peak appeared at 1058 cm^−1^ (peak 9) due to the stretching mode of the C–O–C bonds [50,54].

To elucidate the peptide self-assembly within the alginate ink containing MCC (purple curve), the amide I band region was analyzed. Two shoulders at 1645 cm^−1^ and 1692 cm^−1^ (peaks 10 and 11) were detected, which were also present in the peptide spectrum, by itself (black curve), and are the signatures of the peptide self-assembly via intermolecular hydrogen bonding and carbamate moieties, respectively, corroborating the peptide self-assembly in a β-sheet conformation [55].

### 3.6. In Vitro Cytotoxicity Tests

**Figure 8:**
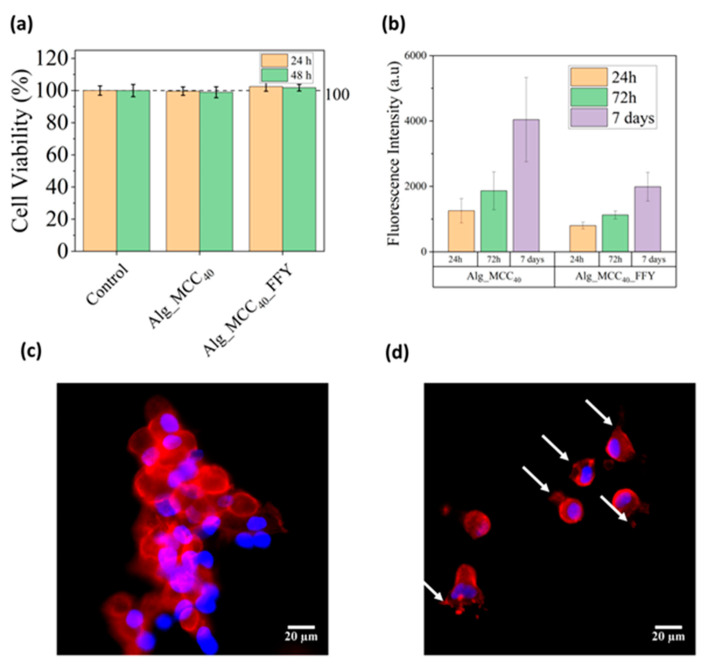
(**a**) The cytotoxicity of the 3D printed scaffolds, Alg_MCC_40_, and Alg_MCC_40__FFY by the MTT indirect test using MG63 cells. (**b**) Alamar Blue assay. (**c**) Fluorescence images of MG63 cells adhered on Alg_MCC_40_. (**d**) Alg_MCC_40__FFY printed scaffolds after 72 h. White arrows point to thee adhesive filopodia bridging cell and material.

The cytotoxicity of the printed scaffolds was studied by an MTT indirect cytotoxicity test (Figure 8a). The results show that the scaffolds do not release cytotoxic components. Moreover, the incorporation of the peptide hydrogel network within the original ink formulation did not produce any cytotoxic effect. In addition, the proliferation of MG-63 cells inside the scaffolds was measured by an AB assay (Figure 8b). It can be observed that the number of cells increased with time, with this increase faster in the scaffolds without the peptide. Moreover, the MG-63 cells were visualized by fluorescence (Figure 8c,d).

As can be observed, the growth in clusters when the peptide is not present indicates that the cell growth is driven by cell-to-cell interactions in a heterogeneous manner, whereas cells are individually and homogenously distributed when the peptide is present in the scaffold. Moreover, focal adhesive filopodia and a more extended shape can be observed when cells are seeded on the Alg_MCC_40__FFY scaffolds. This indicates that the cells are more likely to promote adhesive interactions with the material rather than with other cells, and thus they are in the adhesive stage. The homogeneous distribution of cells in the scaffold and the intimate interaction of the cells with the scaffold are necessary to induce the appropriate cell response and therefore to obtain a functional biomaterial. These results are in agreement with previous works reporting on the modification of alginate hydrogels with cell-affinitive domains such as Arg-Gly-Asp (RGD) to promote the cell–material interactions. In the presence of RGD peptides, cells started to spread after three days as in the case of the Fmoc-FFY reported herein [56]. Moreover, SEM observation of the scaffold cross-sections after three and seven days of culture showed a conserved morphological microstructure with edged particles of microcellulose and cells embedded within the alginate matrix (Figure S5), indicating that the scaffolds were stable in static culture conditions (DMEM, 37 °C pH 7.4).

## 4. Conclusions

In this work, novel alginate–cellulose inks containing a cell-adhesive peptide, Fmoc-FFY, were developed to fabricate biocompatible three-dimensional and shape-defined scaffolds by 3D extrusion printing. Microcrystalline cellulose (MCC) was added to the pre-crosslinked Alginate/Ca^2+^ inks to increase the viscosity and modulate the printability properties. The optimal MCC concentration in the ink formulation for printing, Alg_MCC_40_, was then chosen using the filament and layer stacking tests. The rheological properties of the selected inks including the shear thinning behavior, viscosity, storage modulus, and tan δ were determined by proving that the Alg_MCC_40_ ink possessed better properties for 3D extrusion printing. It was also proven that the incorporation of Fmoc-FFY did not alter the rheological properties and shear thinning behavior of the ink. The 3D scaffolds, printed with the selected ink formulation using the lab-made bioprinter, showed a high shape fidelity, as demonstrated by the SEM. These scaffolds were also characterized by ATR-FTIR spectroscopy confirming the peptide self-assembly within the Alg_MCC_40_ ink by the presence of the characteristic peaks of Fmoc-FFY self-assembly at 1645 and 1692 cm^−1^. Finally, the cytotoxicity properties of the 3D printed scaffolds were assessed in contact with the MG63 osteosarcoma cells, and a homogeneous distribution of the cells on the scaffold was observed, proving their excellent biocompatibility properties for potential bone tissue engineering applications.

## Data Availability

Data sharing is not applicable to this article.

## Supplementary Materials

The following supporting information can be downloaded at: https://www.mdpi.com/article/10.3390/polym14112229/s1, Figure S1: SEM images of MCC particles. Figure S2: SEM images of the top part of the Alg_MCC_40_ scaffolds showing the pore width. Figure S3: Scaffolds prepared with different layer height. Figure S4: 3D printed scaffold. Figure S5: SEM images of the inner part of the scaffolds taken after 3 and 7 days of culture.
