# Supplementary material for: Optimization of the Rheological Properties of Self-Assembled Tripeptide/Alginate/Cellulose Hydrogels for 3D Printing
Source: Polymers (Basel). 2022 May 30;14(11):2229. doi: 10.3390/polym14112229 (PMC9182594; doi:10.3390/polym14112229)
Supplement: Supplementary file 1 [file polymers-14-02229-s001.zip › polymers-1739878-supplementary.pdf]

Supplementary information

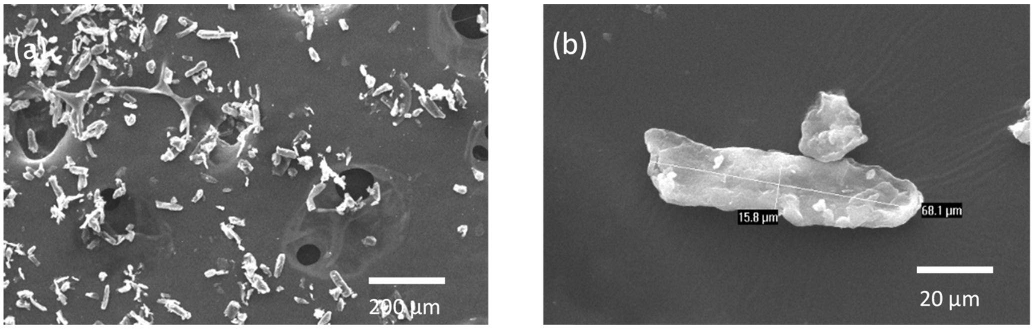

Figure S1. SEM images of MCC particles. **(a)** 100x magnification. **(b)** 1000x magnification.

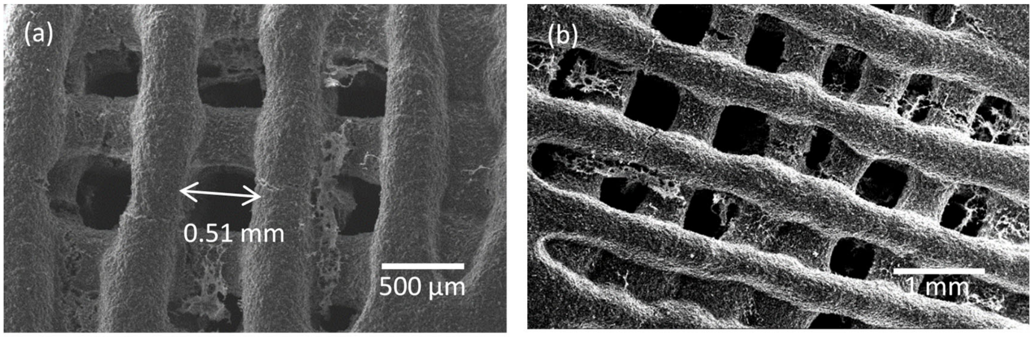

Figure S2. SEM images of the top part of the Alg_MCC$_{40}$ scaffolds showing the pore width. **(a)** 35x magnification. **(b)** 25x magnification.

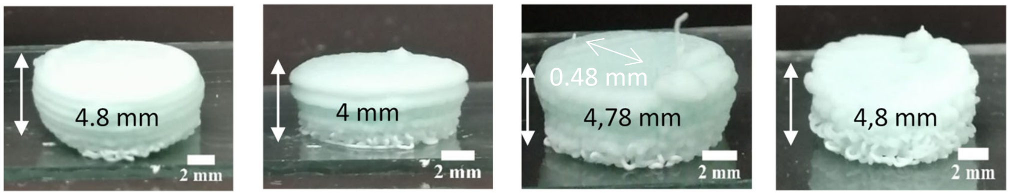

Layer height 0.5 mm Layer height 0.75 Layer height 1 mm Layer height 1.25 mm

Figure S3. Scaffolds prepared with different layer height. Measurements of the total height of the scaffold were taken.

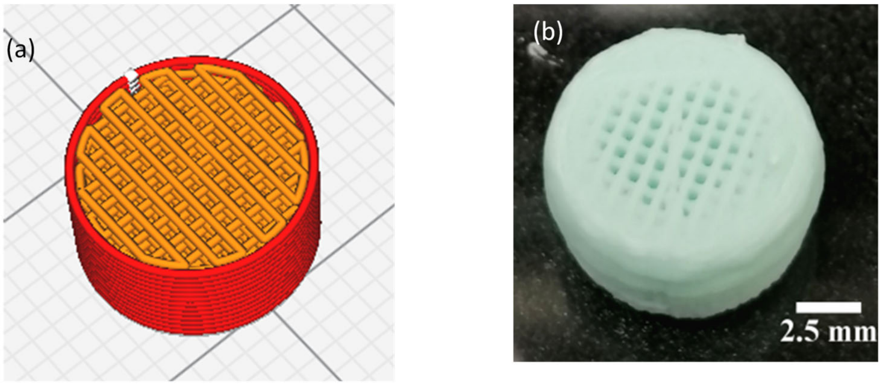

Figure S4. 3D printed scaffold. **(a)** 3D model employed for printing. **(b)** Scaffold obtained from 3D printing.

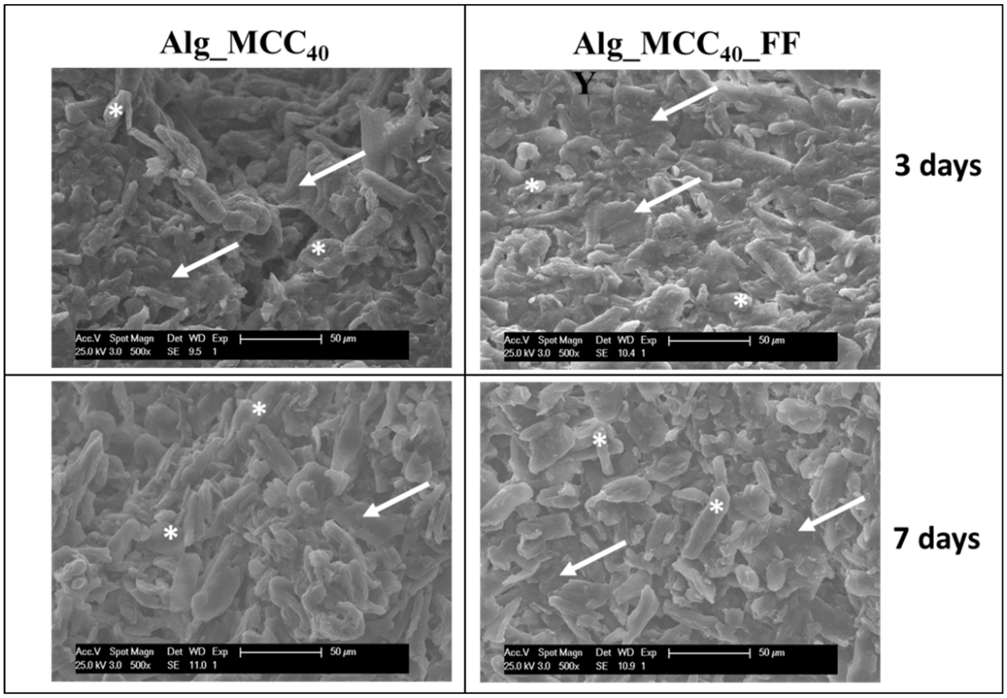

Figure S5. SEM images of the inner part of the scaffolds taken after 3 and 7 days of culture. The images show the characteristic composite microstructure with edged particles of the micro cellulose (white asterisks) and cells (white arrows) embedded within the alginate matrix.
